# Apolipoprotein E isoform-dependent dendritic recovery of hippocampal neurons following activation of innate immunity

**DOI:** 10.1186/1742-2094-3-21

**Published:** 2006-08-25

**Authors:** Izumi Maezawa, Snjezana Zaja-Milatovic, Dejan Milatovic, Christina Stephen, Izabela Sokal, Nobuyo Maeda, Thomas J Montine, Kathleen S Montine

**Affiliations:** 1 Department of Pathology, University of Washington, Seattle, WA, USA; 2 Department of Pathology, University of North Carolina, Chapel Hill, NC, USA

## Abstract

**Background:**

Innate immune activation, including a role for cluster of differentiation 14/toll-like receptor 4 co-receptors (CD14/TLR-4) co-receptors, has been implicated in paracrine damage to neurons in several neurodegenerative diseases that also display stratification of risk or clinical outcome with the common alleles of the apolipoprotein E gene (*APOE*): *APOE2, APOE3*, and *APOE4*. Previously, we have shown that specific stimulation of CD14/TLR-4 with lipopolysaccharide (LPS) leads to greatest innate immune response by primary microglial cultures from targeted replacement (TR) APOE4 mice and greatest p38MAPK-dependent paracrine damage to neurons in mixed primary cultures and hippocampal slice cultures derived from TR APOE4 mice. In contrast, TR APOE2 astrocytes had the highest NF-kappaB activity and no neurotoxicity. Here we tested the hypothesis that direct activation of CD14/TLR-4 *in vivo *would yield different amounts of paracrine damage to hippocampal sector CA1 pyramidal neurons in TR APOE mice.

**Methods:**

We measured *in vivo *changes in dendrite length in hippocampal CA1 neurons using Golgi staining and determined hippocampal apoE levels by Western blot. Neurite outgrowth of cultured primary neurons in response to astrocyte conditioned medium was assessed by measuring neuron length and branch number.

**Results:**

Our results showed that TR APOE4 mice had slightly but significantly shorter dendrites at 6 weeks of age. Following exposure to intracerebroventricular LPS, there was comparable loss of dendrite length at 24 hr among the three TR APOE mice. Recovery of dendrite length over the next 48 hr was greater in TR APOE2 than TR APOE3 mice, while TR APOE4 mice had failure of dendrite regeneration. Cell culture experiments indicated that the enhanced neurotrophic effect of TR APOE2 was LDL related protein-dependent.

**Conclusion:**

The data indicate that the environment within TR APOE2 mouse hippocampus was most supportive of dendrite regeneration while that within TR APOE4 hippocampus failed to support dendrite regeneration in this model of reversible paracrine damage to neurons from innate immune activation, and suggest an explanation for the stratification of clinical outcome with *APOE *seen in several degenerative diseases or brain that are associated with activated innate immune response.

## Background

Innate immune activation has been associated with several neurodegenerative diseases including Alzheimer's disease (AD), Parkinson's disease (PD), amyotrophic lateral sclerosis (ALS), traumatic brain injury, and HIV-encephalitis [1]. Confounding a clear interpretation of the contribution of innate immune activation to neurodegeneration in human diseases and their corresponding animal models is the simultaneous occurrence of multiple pathogenic processes [2]. For this reason, we and others have used a model of selective innate immune activation, intracerebroventricular (ICV) injection of lipopolysaccharide (LPS) [3-10]. LPS specifically activates cluster of differentiation (CD) 14/toll-like receptor (TRL) 4 co-receptors, expressed on glia and especially microglia in brain, with subsequent increase in gene transcription mediated by a required adaptor protein, myeloid differentiation factor 88 (MyD88), and then a bifurcated signaling pathway that is dependent on nuclear factor-kappaB (NF-κB) and p38 mitogen-activated protein kinase (p38MAPK) activities [11,12]. Importantly, a role for CD14/TLR-4 co-receptors is not limited to endotoxemia, as they are important in innate immune response to several endogenous ligands [13], including amyloid beta (Aβ) fibril-stimulated microglial-mediated neurotoxicity [14], as well as peptides and neoantigens expressed by apoptotic cells [15].

We have previously determined for wild-type (wt) C57Bl/6 mice that 24 hrs after LPS treatment there is significant, reversible, free radical damage to neuronal membranes and dendritic degeneration in the absence of febrile response, discernable tissue damage, adaptive immune cell infiltrate, or detectable neuron loss [3]. We investigated changes in both proximal and distal components of the dendritic tree using Golgi staining, which allows direct examination of the dendritic compartment of neurons that is transparent to standard histological techniques, and Sholl analysis, which uses concentric circles centered on the neuron cell body to give a measure of dendritic complexity [16]. We found that hippocampal CA1 pyramidal neurons undergo delayed, reversible spino-dendritic degeneration without neuron death that reaches its nadir at approximately 24 hr and recovers to near basal levels by 72 hr [3]. Furthermore, we have shown that reversible spino-dendritic degeneration in this LPS model is dependent on expression of CD14, TLR4 (but not TLR2), MyD88, the p50 subunit of NF-κB, inducible nitric oxide synthase (iNOS), and the prostaglandin E_2 _receptor EP2 [3,17,18].

Humans, unlike other mammals, have 3 common alleles of the apolipoprotein (apo) E gene (*APOE*), the ε2 (*APOE2*), ε*3 *(*APOE3*), and ε4 (*APOE4*) alleles [19], and inheritance of *APOE4 *is associated with increased risk, earlier onset, or poorer clinical outcome for a number of neurologic diseases that broadly overlap with those associated with innate immune activation cited above: AD, PD, ALS, traumatic brain injury, and HIV-encephalitis [20-28]; at least for AD, inheritance of *APOE2 *also is associated with apparent neuroprotection [29]. While apoE isoform-specific interactions with Aβ peptides likely contribute to the stratification of AD by *APOE*, the mechanisms by which apoE isoforms may influence the clinical outcomes of these several other neurologic diseases is not at all clear. Indeed, results from several groups that investigated aspects of innate immunity have suggested greater response from apoE4- than apoE3-expressing cells or animals [30-33].

ApoE has an established immune modulatory function in the peripheral adaptive immune response to some bacteria and viruses [34]. It also modulates inflammation in cell culture and *in vivo *models of brain injury, where an apoE mimetic therapeutic peptide has been shown to reduce CNS inflammation [35-38]. We have recently published that microglia from targeted replacement (TR) mice that express "humanized" apoE show isoform-dependent innate immune activation from CD14/TLR4 activation and paracrine damage to neurons in mixed primary cultures and hippocampal slices that is least with TR APOE2, intermediate with TR APOE3, and greatest with TR APOE4; these apoE isoform-dependent differences in paracrine damage to neurons are p38MAPK-dependent [39]. Primary astrocyte cultures from these same TR APOE mice show LPS-mediated innate immune activation that is greatest with TR APOE2, less with TR APOE3, and least with TR APOE4 that correlates with NF-κB activity; activated TR APOE2 astrocytes produce the least (none) paracrine damage to neurons among the TR APOE mice [40]. From these cell and organotypic culture data, we hypothesize that apoE isoforms modulate the neurotoxic *vs*. neurotrophic environment following innate immune activation through their differing actions in microglia and astrocytes. This study sought to test that hypothesis *in vivo *by determining whether there are apoE isoform-specific differences in dendritic degeneration and recovery following activation of innate immune response by LPS.

## Methods

### Materials

LPS and RAP (receptor-associated protein) were purchased from Calbiochem (La Jolla, CA). The FD Rapid GolgiStain Kit Stain was purchased from FD Neurotechnologies (Ellicott City, MD). Cell culture media and supplements were purchased from GIBCO (Grand Island, NY). Precast 4–15% SDS-polyacrylamide gels were purchased from BioRad (Hercules, CA). Polyclonal anti-human apoE was purchased from Dako (Carpinteria, CA) and polyclonal anti-MAP2 was purchased from Chemicon International (Temecula, CA). Alexa Fluor^® ^594 goat anti-rabbit secondary antibody was purchased from Invitrogen (Carlsbad, CA). BCA Protein Assay was purchased from Pierce (Rockford, IL).

### Animals

All experiments were performed exactly as approved by the University of Washington IACUC. All mice were female between 5 and 7 weeks of age, were housed at 21 ± 1°C, humidity 50 ± 10%, light/dark cycle of 12 hr/12 hr, and had free access to pelleted food (Rodent Laboratory Chow, Purina Mills, Inc., St. Louis, MO) and water. Homozygous APOE2, APOE3 and APOE4 targeted replacement (TR) mice 'humanized' at apoE were developed by Dr. Maeda and colleagues and are backcrossed greater than six generations to a C67Bl/6 genetic background [41,42]. Briefly, all three lines contain mouse *apoE *5' regulatory sequences continuous with mouse exon 1 (noncoding) followed by human *APOE *exons (and introns) 2–4. ICV LPS or vehicle (phosphate-buffered saline) was delivered exactly as previously described [5]. Briefly, following anesthesia, a 5 μl ICV injection was delivered over 1 minute into the left lateral ventricle. LPS was dissolved in PBS (pH 7.4) at a final concentration of 1 mg/ml for a total ICV dose of 5 μg. Animals were euthanized and the ipsilateral cerebral hemisphere was removed and either cut in two and immediately immersed in Golgi impregnation solution, or the ipsilateral hippocampus dissected out, flash-frozen in liquid N_2_, and stored at -80°C for western blotting. Sixteen mice with each TR APOE were used: eight exposed to LPS and eight PBS controls. These were then stratified to sacrifice at 24 or 72 hr (n = 4 in each group) and these equally divided between morphometric and biochemical assays. We have previously shown that ICV saline injection results in no change in LPS-sensitive endpoints such as F_2_-isprostane, F_4_-neuroprostane and citrulline levels [5].

### Morphometric measurement

Golgi staining of 50 micron thick sections from paraffin-embedded blocks was as previously described by us [3] following the manufacturer's specifications (FD Rapid GolgiStain Kit). Eight mice from each genotype were processed for Golgi staining: four exposed to LPS and four exposed to PBS. These were divided equally between the two time points to give two mice per genotype, exposure, and time point. Six Golgi-impregnated pyramidal neurons with no breaks in staining along the dendrites from CA1 sector of hippocampus were randomly selected from each mouse and traced according to the methods previously described by us using Neurolucida (MicroBrightField, Williston, VT) at ×1000 [3]. SZM and CS were blinded to *APOE *and treatment (LPS or saline) of each section. Dendrite length was calculated by Neuroexplorer (MicroBrightField) utilizing the Sholl method of concentric circles [16] and is presented as total dendrite length (μm) per Sholl compartment.

### Western blotting of hippocampal apoE

Hippocampal homogenates were prepared by the addition of 200 μl of lysis buffer (60 mM Tris pH6.8, 2% SDS, 10% glycerol) to each frozen ipsilateral hippocampus (~5 mg) followed by sonication, boiling, and centrifugation to remove insoluble debris. The resulting homogenate was assayed for protein concentration and 10 μg of each sample separated by gel electrophoresis, transferred and western blotted as previously described by us [43]. Signal was developed by enhanced chemiluminescence (Amersham, Piscataway, NJ) and band intensity analyzed by scanning densitometry using ImageJ software to determine background-corrected absorbance units.

### Astrocyte conditioned medium (CM)

Preparation of primary cultures of 1-day-old mouse cerebral cortical astrocytes from TR APOE mice was as described previously [43]. Briefly, 1 week-old astrocytes (1 × 10^6 ^cells/T75 flask) were washed with PBS and cultured with serum-free Neurobasal medium containing 125 mM glutamine and B27 supplement (1 ml/50 ml) for 72 hrs to generate astrocyte CM.

### Neurite outgrowth

Primary cortical neurons were derived from the cerebral cortex of newborn mice following the protocol of Xiang *et al*. [44] as previously done by us [39]. 10^5 ^cells were plated onto poly-D-lysine coated coverslips and used after 10 days *in vitro *(DIV). 10 DIV neurons were incubated with 50% astrocyte CM (see above) and 50% Neurobasal medium or 100% Neurobasal medium (control) for 48 hrs. At this time, cells were rinsed, fixed, and prepared for immunocytochemistry as previously described [43]. Cells were incubated overnight with MAP2 antibody (1:500), washed, and incubated with Alexa Fluor^® ^594 goat anti-rabbit secondary antibody (1:1000) for 2 hr. The cells were washed and mounted in Vectashield mounting media (Vector Lab, Burlington, CA) containing 4'-6-diamidino-2-phenylindole (DAPI) for nuclear label. Images were examined with a confocal microscope using LaserSharp software (BioRad). Five or more MAP2-stained neurons were randomly selected and dendrite length and branch number measured with Neurolucida. SZM and CS were blinded to *APOE *of the astroctyes used to generate CM.

## Results

**Figure 1:**
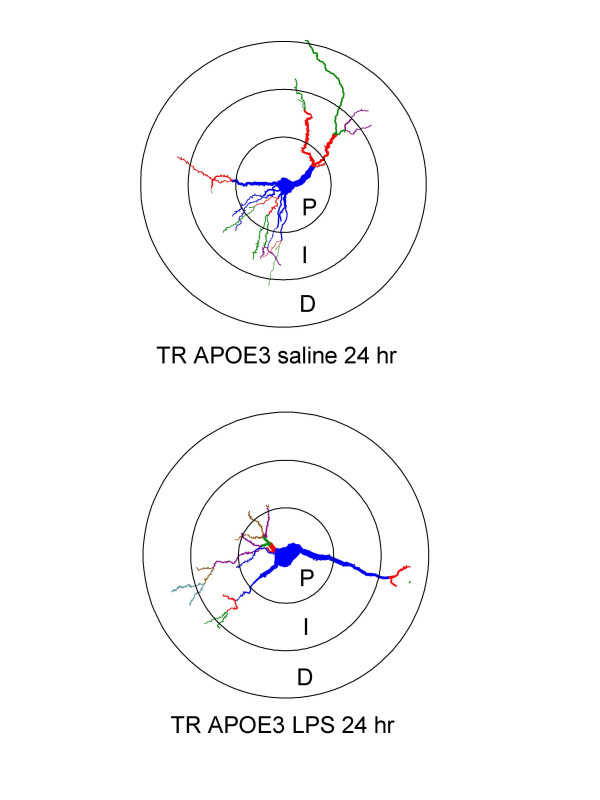
**Representative neurons 24 hrs after treatment with saline or LPS**. Neurolucida tracings of two CA1 hippocampal pyramidal neurons stained by Golgi method; blue is soma and first order dendrites, red is second order dendrites, green is third order dendrites, purple is fourth order dendrites. Sholl compartments are indicated by circles that represent 0–50 μm (P: proximal), 51–100 μm (I: intermediate), and 101–150 μm (D: distal) from the center of the soma. For the two neurons shown here, saline had total dendrite lengths of 614.1 (P), 617.3 (I), and 64 (D) microns as calculated by Neuroexplorer. LPS had dendrite lengths of 382.3 (D), 321.4 (I), and 35.9 (D) microns. Data for all neurons examined are shown in the Table.

Six week-old TR APOE mice were ICV injected with LPS or vehicle (phosphate-buffered saline) and sacrificed after 24 or 72 hrs. Ipsilateral cerebral hemispheres were removed and the hippocampal CA1 region Golgi stained for morphometric analysis as previously described by us [3]. Figure 1 shows representative Neurolucida images for two pyramidal neurons (with or without LPS) used to calculate total dendrite length for each Sholl compartment. The Table summarizes our results for the three genotypes (n = 8 to 12 neurons examined for each condition and genotype) at 24 hrs. Our results for saline treatment showed that there was a significant difference in basal dendrite length in the distal (101–150 μm) Sholl compartment of CA1 pyramidal neurons among the TR APOE mice in the absence of a specifically applied stress with reduced length in TR APOE4 mice. Because of this variation, data for ICV LPS-induced changes after 24 hrs were analyzed as the percentage of basal values for that TR APOE line (Table). Similar to our previous findings with wt mice [3,17,18], dendrite length was significantly reduced in all TR APOE mice in all three compartments 24 hrs after LPS treatment. We found no difference in the extent of reduction among genotypes, with two-way ANOVA showing no significant difference in decrease in dendrite length among TR APOE in any of the Sholl compartments at 24 hr post-ICV LPS.

**Figure 2:**
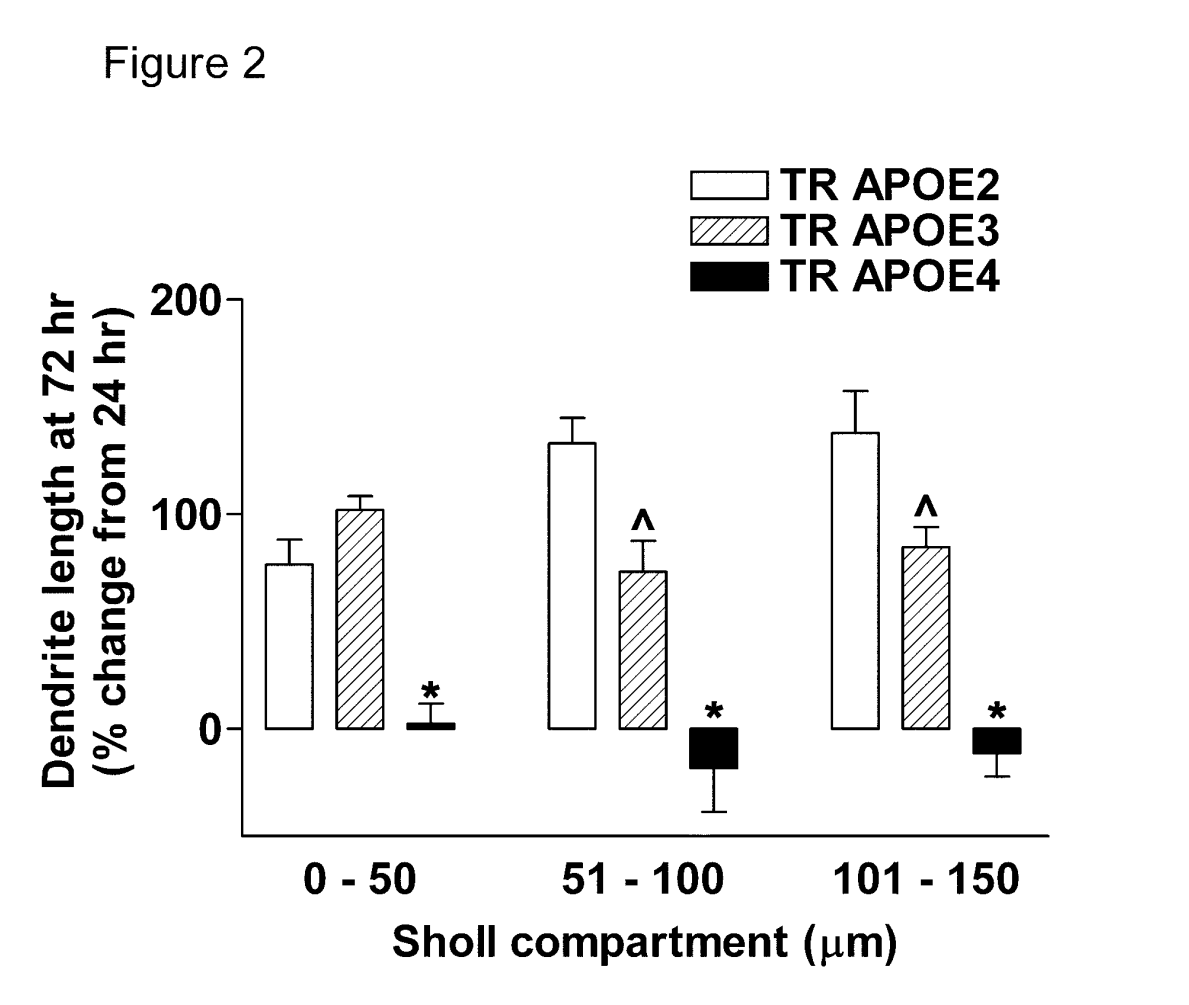
**Dendrite length recovery 72 hours after LPS**. Data are total dendrite length in each Sholl compartment of hippocampal CA1 pyramidal neurons from TR APOE mice 72 hr post ICV LPS expressed as percent change from 24 hr post-ICV LPS for each genotype. n = 12 neurons for each group (6 neurons per mouse for each genotype, time point, and exposure). Two-way ANOVA for recovery of dendrite length had P < 0.0001 for TR APOE but P > 0.05 for Sholl compartment. In the proximal Sholl compartment, one-way ANOVA had P < 0.0001 for % change in dendrite length among TR APOE; Bonferroni-corrected repeated pair analysis had *P < 0.001 for TR APOE2 or TR APOE3 *vs*. TR APOE4 but ^P > 0.05 for TR APOE2 *vs*. TR APOE3. In both the intermediate and distal Sholl compartments, one-way ANOVA had P < 0.0001 for % change in dendrite length among TR APOE; Bonferroni-corrected repeated pair analysis had *P < 0.001 for TR APOE2 or TR APOE3 *vs*. TR APOE4 and ^P < 0.05 for TR APOE2 *vs*. TR APOE3.

We have previously found in wt mice that following ICV LPS injection, several indices of neuronal damage, including morphometric measures, have returned to near basal levels by 72 hr [3]. We compared recovery at 72 hrs among the three genotypes and found a striking difference in the recovery of TR APOE4 in all 3 Sholl compartments (Figure 2). Similar to our previous wt results, dendrite length of neurons in both TR APOE2 and TR APOE3 mice recovered extensively in all 3 compartments, with TR APOE2 recovery better than TR APOE3 (P < 0.05 in the intermediate and distal Sholl compartments). In contrast, dendrite length of pyramidal neurons in TR APOE4 mice did not show any recovery in any of the compartments (P < 0.001).

**Figure 3:**
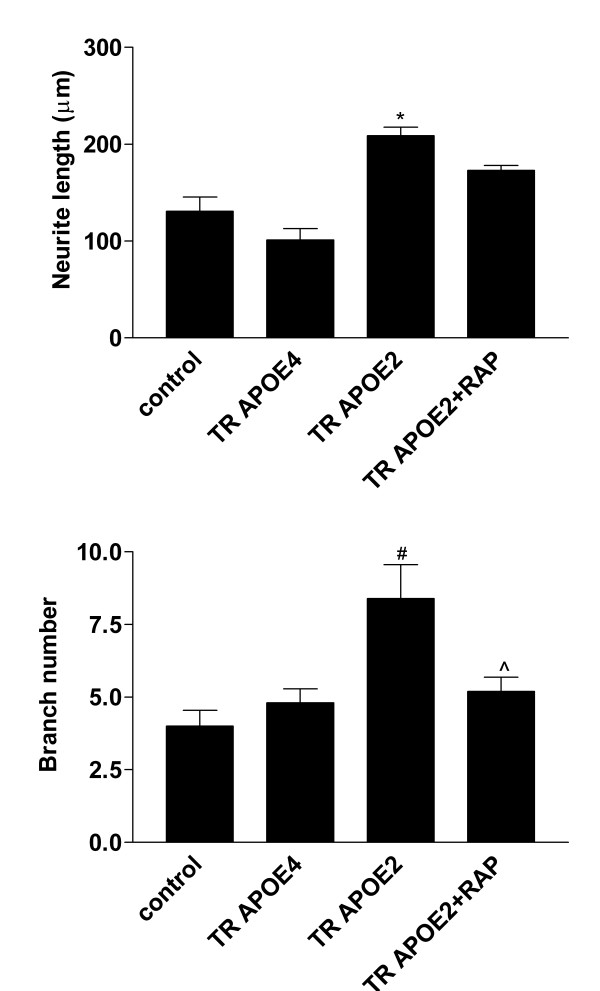
**Neurite outgrowth following incubation with astrocyte conditioned medium (CM)**. CM was generated from TR APOE primary astrocytic cultures and collected after 72 hours. Primary wt neurons were treated with astrocyte CM (50% of total volume) or 100% Neurobasal medium (control). For RAP treatment, neurons were preincubated with 200 nM RAP for 2 hrs prior to the addition of CM. After 48 hours, neurons were immunostained with anti-MAP2 antibody (1:500) and examined with a confocal microscope using LaserSharp software (BioRad). Five or more MAP2-stained neurons were randomly selected and dendrite length (**A**) and branch number (**B**) determined by Neurolucida. Data are expressed as mean length or branch number ± SEM (n = 5). One-way ANOVA for neurite length (A) had P < 0.0001 and Bonferroni-corrected repeated pair analysis had *P < 0.001 for TR APOE2 *vs*. control but P > 0.05 for TR APOE4 or TR APOE2+RAP *vs*. control. For branch number (B), one-way ANOVA had P < 0.05 and Bonferroni-corrected repeated pair analysis had ^#^P < 0.01 for TR APOE2 *vs*. control, P > 0.05 for TR APOE4 or TR APOE2+RAP *vs*. control, and ^P < 0.05 for TR APOE2 *vs*. TR APOE2+RAP.

Dr. Maeda and colleagues have previously demonstrated that despite brain regional differences in apoE expression, there are no isoform-specific differences in levels of apoE in brain tissue from these TR APOE mice, even with elevated apoE2 plasma levels [45]. We have also previously found no difference in apoE concentration in CM from cultured astrocytes or microglia from the three genotypes with or without LPS treatment [39,40,43]. To confirm similar basal levels of apoE and determine the effects (if any) of LPS, we performed Western blot analysis of hippocampal homogenates from parallel ICV-injected mice and compared apoE levels among the three TR APOE mice 24 and 72 hrs after saline or LPS ICV injection. There was no significant difference in cerebral apoE levels among the three TR APOE mice, and no effect of LPS on apoE levels 24 or 72 hrs after LPS activation. We also compared the effects of conditioned medium (CM) from TR APOE2 and APOE4 astrocytes on neurite length and branch number in primary neuronal cultures, since these measurements are comparable to our morphometric *in vivo *data. We incubated primary cultures of wt cerebral neurons with CM from primary cultures of TR APOE2 or TR APOE4 astrocytes and found that TR APOE2 astrocyte CM supported significant neurite growth while TR APOE4 astrocyte CM had no apparent effect on neurite length or branch number (Figure 3). Since we have previously ruled out differences in apoE protein levels in astrocyte CM among the TR APOEs [40,43], we turned to apoE receptor binding as a mechanistic basis for this difference. We tested the effect of an apoE receptor antagonist, receptor-associated protein (RAP), on neurite stimulation from TR APOE2 astrocyte CM by preincubating neuron cultures with a concentration (200 nM) of RAP that specifically inhibits the LDL receptor-related protein (LRP) [46-48]. RAP treatment reduced the effect of TR APOE2 astrocyte CM so that it no longer had a significant effect on either neurite length or branch number (Figure 3).

## Discussion

Several investigators have reported direct neurotrophic and neurotoxic actions of recombinant or purified apoE isoforms using a variety of culture systems (reviewed in [49]). In addition to these direct effects on neurons, we have recently published that glia from TR APOE mice show apoE isoform-dependent innate immune activation following stimulation of CD14/TLR4 co-receptors with LPS [39,40]. Specifically, our experiments have shown greater innate immune response by TR APOE4 microglia and greater p38MAPK-dependent paracrine damage to neurons in mixed primary cultures and hippocampal slice cultures from TR APOE4 mice. In contrast, TR APOE2 astrocytes had the highest NF-κB activity and least (none) neurotoxicity following innate immune activation. Thus, in addition to the direct effects of apoE on neurons, our cell culture data show that apoE isoform-dependent actions in glia may also alter paracrine effects of astrocytes and microglia on neurons and thereby contribute to neurotoxic *vs*. neurotrophic outcomes associated with inheritance of different APOE alleles. Our *in vivo *results presented here are consistent with this model and further demonstrate that neurite repair following paracrine damage from innate immune activation is modulated by TR APOE and that the neurotrophic actions of TR APOE2 CM may be largely RAP-dependent.

Several laboratories have investigated apoE isoform-specific neurotrophic actions in a variety of cell culture models. The initial studies were performed using fetal rabbit dorsal root ganglion cultures and showed that, in the presence of beta-migrating very low density lipoproteins, apoE3 increased neurite outgrowth whereas apoE4 decreased neurite outgrowth [50]. Using immortalized and transfected neuronal cell line, subsequent investigations confirmed the neurotrophic effects of apoE3 relative to apoE4 and showed that the neurotrophic effect of apoE3 was dependent on interaction with the heparin sulfate proteoglycan-low density lipoprotein receptor-related protein (LRP) pathway [51-54] when incorporated into several different lipid particles and even without the addition of exogenous lipid [55,56]. The next series of experiments used primary cultures derived from transgenic mice that express apoE3 or apo4 under the glial fibrillary acidic protein promoter. Again, these showed greater relative neurotrophism of apoE3 expression compared to apoE4 that was largely LRP-dependent [57,58]. Using these same transgenic mice aged 1 to 2 years, a final *in vivo *study demonstrated greater spine density in transgenic mice expressing human apoE3 than human apoE4 [59]. It is important to note that while all of these studies support the proposal that apoE4 expression could underlie relative regenerative failure of injured central nervous system neurons, none have investigated the relative neurotrophic actions of apoE2.

Our *in vivo *results with TR mice are consonant with these cell culture studies and agree with the single other *in vivo *study that used transgenic mice by indicating a relatively more neurotrophic environment in the presence of apoE3 *vs*. apoE4. We add to this knowledge by showing that expression of apoE2 also supports a more neurotrophic environment than apoE4. Indeed, our experiments with ICV LPS provide *in vivo *support for the proposal made from cell culture data that apoE4 may play a role in neurite regenerative failure [58]. Our observations on apoE2 go further because we observed enhanced neurite regeneration in TR APOE2 *vs*. TR APOE3 mice; this is a novel observation that resonates with genetic association studies that indicate that inheritance of APOE2 reduces the risk of AD. Like the enhanced neurotrophic action of apoE3, our cell culture data suggest that the greater neurotrophic actions of apoE2 may derive, at least in part, from astrocyte-secreted apoE2 and may be dependent on apoE2-LRP interaction. However, conditioned medium from primary astrocytes is an artificial system with many active factors and so these results must be interpreted with caution.

In summary, our results from TR APOE mice that express each of the common apoE isoforms indicate a relatively least neurotrophic environment in TR APOE4 mice compared to TR APOE3 or TR APOE2 under basal conditions. Moreover, following reversible paracrine damage to neurons from direct activation of CD14/TLR4 receptor there was failure of neurite regeneration in TR APOE4 mice and greater regeneration in TR APOE2 mice compared to TR APOE3 mice. The observations offer an explanation for the stratification of clinical outcome with APOE seen in several degenerative diseases or brain that are associated with activated innate immune response.

## Conclusion

The data indicate that the environment within TR APOE2 mouse hippocampus was most supportive of dendrite regeneration while that within TR APOE4 hippocampus failed to support dendrite regeneration in this model of reversible paracrine damage to neurons from innate immune activation, and suggest an explanation for the stratification of clinical outcome with *APOE *seen in several degenerative diseases or brain that are associated with activated innate immune response.

## Abbreviations

AD: Alzheimer's disease; ALS: amyotrophic lateral sclerosis; apo: apolipoprotein; Aβ: amyloid beta; CD: cluster of differentiation; CM: conditioned medium; DAPI: 4'-6-diamidino-2-phenylindole ; DIV: days *in vitro*; ICV: intracerebroventricular; iNOS: inducible nitric oxide synthase; LPS: lipopolysaccharide; LRP: LDL receptor-related protein; MAP2: microtubule-associated protein 2; MyD88: myeloid differentiation primary response protein; NF-κB: nuclear factor kappaB; p38MAPK: p38 mitogen-associated protein kinase; PD: Parkinson's disease; RAP: receptor-associated protein; TLR: toll-like receptor; TR: targeted replacement; wt: wild type.

## Competing interests

The author(s) declare that they have no competing interests.

## Authors' contributions

IM, SZM, DM, CS and IS performed the experiments described. NM developed the mouse line that was used in all experiments. TJM conceived the study and its design and helped to draft the manuscript. KSM analyzed the data, prepared the figures, and drafted the manuscript.

**Table 1:** Dendrite length 24 hrs after ICV injection of saline or LPS.

	**apoE2**		**apoE3**		**apoE4**	
	
	saline	LPS	Saline	LPS	saline	LPS
*Sholl r (μm)*	*microns*	*% of saline*	*microns*	*% of saline*	*microns*	*% of saline*

0–50	523 ± 38	52.9 ± 5.1	497 ± 48	44.7 ± 3.3	542 ± 36	44.6 ± 4.7
51–100	497 ± 36	40.1 ± 4.9	456 ± 47	36.8 ± 10.0	513 ± 39	36.9 ± 7.2
101–150	98 ± 13	42.2 ± 10.4	80 ± 10	30.0 ± 10.1	**58 ± 8^**	33.1 ± 9.8

Total dendrite length for each Sholl compartment for mouse pyramidal neurons within hippocampal sector CA1 was determined by Golgi staining followed by analysis with Neurolucida. Data are from the three lines of TR APOE mice with four mice from each genotype examined at this time point. For PBS-treated mice (n = 6 neurons per mouse in each group for a total of 12 neurons per genotype and exposure), two-way ANOVA had P < 0.0001 for Sholl compartment but P > 0.05 for TR APOE; however, one-way ANOVA in the distal Sholl compartment of the three genotypes had P < 0.05 and Bonferroni-corrected repeated pair comparisons had ^P < 0.05 for TR APOE2 *vs*. TR APOE4 but no other paired comparisons. For ICV LPS-exposed mice, two-way ANOVA was not significant for TR APOE or Sholl compartment at 24 hr post ICV LPS (n = 6 neurons per mouse in each group for a total of 12 neurons per genotype and exposure), nor was one-way ANOVA across any of the Sholl compartments. These values encompass our previous results with ICV LPS in C57Bl/6 mice [3, 17, 18]
